# Changes in the Level of DNA Methylation in *Candida albicans* under the Influence of Physical and Chemical Factors

**DOI:** 10.3390/ijms242115873

**Published:** 2023-11-01

**Authors:** Magdalena Gryzinska, Barbara Kot, Ewa Dudzinska, Anna Biernasiuk, Andrzej Jakubczak, Anna Malm, Katarzyna Andraszek

**Affiliations:** 1 Institute of Biological Basis of Animal Production, University of Life Sciences in Lublin, 20-950 Lublin, Poland; andrzej.jakubczak@up.lublin.pl; 2 Institute of Biological Sciences, University of Siedlce, 08-110 Siedlce, Poland; 3 Department of Dietetics and Nutrition Education, Medical University of Lublin, 20-093 Lublin, Poland; ewa.dudzinska@umlub.pl; 4 Chair and Department of Pharmaceutical Microbiology, Medical University of Lublin, 20-093 Lublin, Poland; anna.biernasiuk@umlub.pl (A.B.); anna.malm@umlub.pl (A.M.); 5 Institute of Animal Science and Fisheries, University of Siedlce, 08–110 Siedlce, Poland; katarzyna.andraszek@uph.edu.pl

**Keywords:** epigenetics, methylation DNA, infrared radiation, microwave treatment, UVC, X-ray radiation, hypoxia, increased CO_2_ concentration in the atmosphere

## Abstract

The effects of physical factors such as radiation (electromagnetic, microwave, infrared, laser, UVC, and X-ray) and high temperature, as well as chemical factors (controlled atmosphere) on the level of global DNA cytosine methylation in *C. albicans* ATCC 10231 cells were investigated. Prolonged exposure to each type of radiation significantly increased the DNA methylation level. In addition, the global methylation level in *C. albicans* cells increased with the incubation temperature. An increase in the percentage of methylated DNA was also noted in *C. albicans* cells cultured in an atmosphere with reduced O_2_. In contrast, in an atmosphere containing more than 3% CO_2_ and in anaerobic conditions, the DNA methylation level decreased relative to the control. This study showed that prolonged exposure to various types of radiation and high temperature as well as reduced O_2_ in the atmosphere caused a significant increase in the global DNA methylation level. This is most likely a response protecting DNA against damage, which at the same time can lead to epigenetic disorders, and in consequence can adversely affect the functioning of the organism.

## 1. Introduction

DNA methylation is an epigenetic process. It is associated with genetics, as a ‘second code’ determining the activity or silencing of genes in the cell. Epigenetics is the study of the effect of the environment on changes in gene expression taking place during the life of an individual. Expression modified by external factors can be inherited. Epigenetic processes respond to stimuli from the environment and change the expression of genes in order to adapt the organism to the environmental conditions [1,2]. In addition to DNA methylation, epigenetic mechanisms include histone modifications, gene silencing based on miRNA, and chromatin remodeling [3].

DNA methylation is a chemical modification of DNA involving the addition of a methyl group to a DNA molecule, which takes place mainly at cytosine–guanine dinucleotide (CpG) sites [4]. Methyl groups are transferred by DNA-methyltransferases (DNMTs). Methylated cytosine is an important epigenetic factor enabling the regulation of gene expression. Active transcription regions of the genome have low methylated cytosine content, while the presence of methylated CpG within gene promoters can result in the inhibition of gene transcription or expression, as well as a loss of function in the gene products [5].

DNA methylation is an important epigenetic marker that plays important roles in orchestrating gene expression and maintaining genome stability. DNA methylation is involved in inhibiting gene transcription, silencing repetitive elements of the genome, and inactivating the X-chromosome [6]. The content of 5mC in eukaryotic genomes is highly varied. In mammalian cells, the percentage of methylated cytosine is ~3–8% of total cytosine, while the percentage in plants can be as high as 30%. The most sensitive method used to analyze the content of 5mC DNA requires the methylation of at least 0.075% of the total cytosine. It is possible that a low level of 5mC in yeast genomes makes it impossible to detect. However, the DNA methylation level of 0.3–1% reported by Hattman et al. [7] was much higher than the limit of detection in HPLC or digestion with restriction enzymes. This suggests that these methods should theoretically be able to detect 5mC if the genome of yeast contains 0.3–1% 5-methylcytosine.

Every species has its own characteristic, strictly defined methylation sites, which constitute patterns enabling species identification. These sites undergo major changes during both embryonic and postnatal development [8]. The methylation level is currently treated as an indicator of the age and lifespan of organisms. Disorders of this process can be the cause of premature ageing [9,10]. In addition to DNA methylation, this process can also affect the entire chromatin. Histone proteins, which build nucleosomes, undergo a variety of biochemical processes, including methylation. In addition, these proteins are highly conserved—their homologous domains have been detected in mammals, plants, and fungi [11,12].

Individual epigenetic mechanisms influence one another. Their interactions are not yet well known, but an increasing number of studies on the interactions between DNA methylation, histone modifications, and miRNA modifications continue to improve our understanding of how their combined action regulates gene activity. Thus far, it has been demonstrated that changes in the DNA methylation pattern are usually the consequence, and not the cause, of other epigenetic changes. A relationship has been observed between the DNA methylation level and the transcription activity of a given DNA fragment. Hypermethylation (an elevated methylation level) is characteristic of heterochromatin and silenced euchromatin regions, whereas hypomethylation (a reduced methylation level) is often observed in transcriptionally active genes [13].

Research conducted in order to identify environmental factors affecting the degree of DNA methylation and understand epigenetics can be helpful in conservation measures aimed at the survival of species. For example, research aimed at identifying species sensitive to environmental changes and those which will tolerate them can prove invaluable for preserving current ecosystems. Furthermore, epigenetic studies on genes involved in adaptation to various changing environmental conditions can help in planning selective breeding or improving genetic engineering methods. Examples of epigenetic processes in yeast are inheritance, associated with the presence of prions, and the control of the transposition of transposons and retrotransposons through DNA methylation [14]. In yeast cells, the structure of chromatin without histone deacetylase activity exhibits the early activation of late-replicating ori sequences located in the internal chromosomal loci [15].

In nearly all higher eukaryotes, from yeast to plants and mammals, the functional relationship between DNA methylation and post-translational histone modifications is crucial to the stabilization of nucleosomes and the formation of heterochromatin [16]. In the yeast *Saccharomyces pombe*, the inactivation of this machinery has led to the global disruption of gene expression and the loss of H3K9 methylation, resulting in multiple incorrect transcripts [17]. Studying the impact of environmental factors in order to establish which of them can significantly influence the degree of DNA methylation, which leads to changes in gene expression, can provide valuable information on their effects on living organisms. Due to the limitations of in vivo research, studies are conducted using model organisms with similar biological traits. Microorganisms that can be used as a model organism in epigenetic research include the polymorphic fungus *Candida albicans*. In studies assessing the effect of physical and chemical factors on the degree of DNA methylation, this microbe can be used to identify the factors with the greatest effect on the DNA of eukaryotes. It can also provide a basis for research aimed at understanding how the degree of DNA methylation influences the expression of genes associated with the production of virulence factors by this microbe.

*C. albicans* is a human commensal colonizing diverse niches, such as the skin and the urogenital and gastrointestinal tracts. In most people, *C. albicans* resides as a lifelong, harmless commensal [18]. In immunocompromised patients, however, including diabetics, organ transplant recipients, and HIV-positive individuals, *C. albicans* may cause various chronic or acute opportunistic infections, including thrush, nail lesions, cutaneous infections, vaginitis, endocarditis, meningitis, encephalitis, arthritis, and pyelonephritis [19]. Together with the increase in the number of therapeutic options, including immunosuppression therapy, intensive anticancer therapy, and treatment with broad-spectrum antibiotics, as well as the increasing number of patients with various systemic diseases impairing cellular immunity (e.g., diabetes), the frequency of invasive mycoses caused by *C. albicans* has increased as well [20,21]. The switch from the commensal phase to the pathogenic phase of *C. albicans* may be associated with phenotypic plasticity. *C. albicans* can grow in several morphological forms, including the unicellular yeast form, elongated hyphae, and pseudohyphae [22]. The ability to switch between yeast and filamentous forms is thought to be closely linked to virulence. Filamentous cells are more invasive and more easily penetrate tissues, while yeast cells are easily disseminated in the bloodstream [20].

The aim of this study was to assess the effect of selected physical and chemical factors (various types of radiation, temperature, reduced O_2_ concentration, lack of O_2_ in the atmosphere, and increased CO_2_ concentration) on the level of the global cytosine methylation of DNA in cells of the fungus *C. albicans*, which is part of the human microbiota and has been used as a model eukaryotic organism. Finding the factors that most significantly increase the level of global DNA methylation will allow future studies using other eukaryotic model organisms to assess the impact of these factors on the level of methylation and the expression of selected genes, e.g., oncogenes or genes associated with chronic inflammation, which may be helpful in finding a factor with potential as a therapeutic tool.

## 2. Results

The global level of DNA methylation in *C. albicans* ATCC 10231 cells exposed to selected physical factors is presented in Table 1.

**Figure 1:**
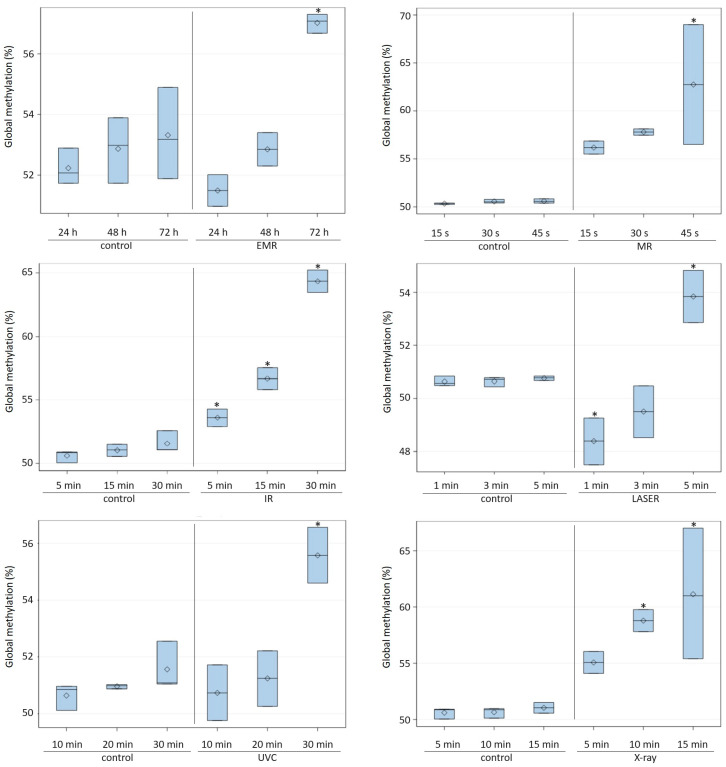
DNA methylation level (%) in *Candida albicans* ATCC 10231 cells exposed to various types of radiation for various time periods. EMR—electromagnetic radiation, MR—microwave radiation, IR—infrared radiation, LASER—Light Amplification by Stimulated Emission of Radiation; UVC—ultraviolet radiation in a wavelength range of 100–280 nm (C); X-ray—roentgen radiation. Asterisks indicate significant differences in DNA methylation levels at P ≤ 0.05 between the test and control samples (Duncan’s test).

**Figure 2:**
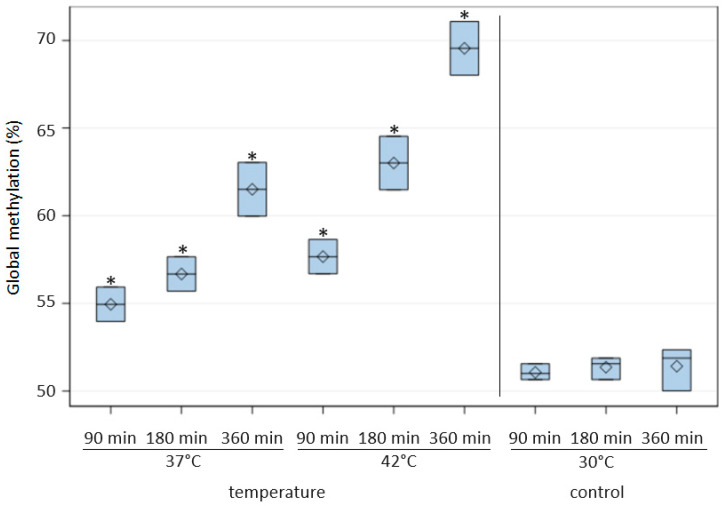
DNA methylation levels (%) in *Candida albicans* ATCC 10231 cells incubated at elevated temperatures (37 °C and 42 °C) for various time periods. Asterisks indicate significant differences in DNA methylation levels at P ≤ 0.05 between the test and control samples (Duncan’s test).

**Figure 3:**
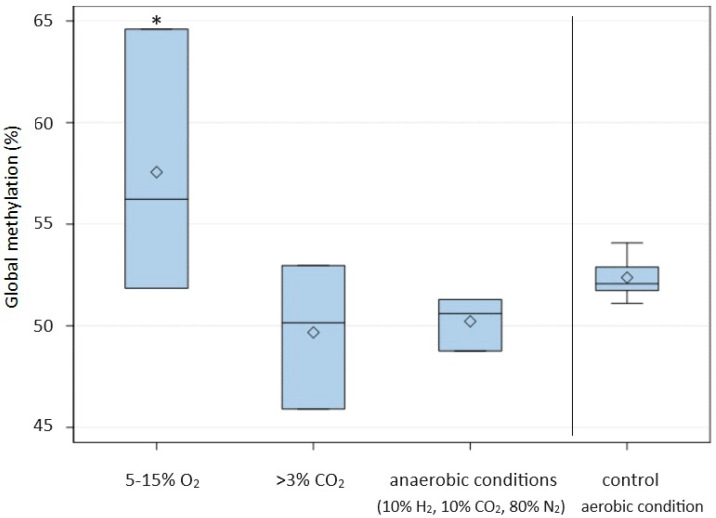
DNA methylation level (%) under the influence of changes in the content of O_2_ and CO_2_ in the atmosphere in which *Candida albicans* ATCC 10231 was cultured in a 24 h period. Asterisks indicate significant differences in DNA methylation levels at P ≤ 0.05 between the test and control samples (Duncan’s test).

The results showed that irrespective of the type of radiation used, the level of DNA methylation increased with the exposure time (Figure 1). The highest percentage of methylated DNA in *C. albicans* was obtained after 30 min of exposure to IR (64.36 ± 0.88) and 45 s of exposure to MR (62.75 ± 6.24). The average percentage of methylated DNA in *C. albicans* after IR treatment was significantly (*p* ≤ 0.05) higher than in cells not exposed to this radiation and also significantly higher at successive time points (Table 1). A high percentage of methylated DNA in *C. albicans* was also found following X-ray radiation after 10 (58.79 ± 0.98) and 15 min (61.14 ± 5.81), and these methylation levels were significantly higher than in the control groups. A significant increase in DNA methylation compared to the control was also observed after the prolonged exposure of *C. albicans* cells to other types of radiation (LASER, EMR, and UVC) (Figure 1). When *C. albicans* cells were exposed to LASER for a short time (1 or 3 min), the level of global methylation was lower than in non-irradiated cells. The DNA methylation levels of *C. albicans* cells incubated for 90, 180, and 360 min at 37 °C and 42 °C were significantly higher than the level in cells incubated for the same time at 30 °C (control) (Figure 2). In addition, extending the incubation time under these conditions resulted in an increase in the level of methylation, with the highest average percentage of methylated DNA (69.55 ± 1.53) noted after 360 min at 42 °C (Figure 2). The cultivation of *C. albicans* for 24 h in conditions of reduced oxygen (5–15% O_2_) resulted in a significant increase in the global methylation level. The average percentage of methylated DNA (57.56 ± 6.48) in these conditions was significantly higher than in the control conditions (aerobic environment), in anaerobic conditions, and in the presence of more than 3% CO_2_ (Table 1, Figure 3).

## 3. Discussion

Yeasts are widely used as model organisms for studying cellular metabolism, the regulation of the cell cycle, and signal transduction. However, it remains controversial whether methylated cytosine (5-methylcytosine, 5mC) exists in the yeast genome. Tang et al. [23] developed a sensitive method based on gas chromatography/mass spectrometry (GC/MS) and studied the incidence of 5mC in 19 strains of yeast representing 16 species. The results indicated that DNA methylation is widespread in yeast, with DNA methylation in the entire genome of the test strains of yeast ranging from 0.014% to 0.364%, i.e., only one to two orders of magnitude lower than in mammalian cells (3–8%). However, the global DNA methylation level in *C. albicans* in this study is high (>50%) because of the storage and cultivation of C. *albicans* under laboratory conditions. Turchetti et al. (2020), similarly to us, also obtained high levels of DNA methylation under control conditions in the tested yeast species (the average levels of DNA methylation in *Naganishia antarctica* and *N. albida* were 70.97% and 58.3%, respectively) [24]. It was demonstrated that the content of 5mC in yeasts differed significantly at different stages of growth and that the DNA methylation inhibitor 5-azacytidine can cause a decrease in DNA methylation in the entire genome, as in mammalian cells. The demonstration of the universal presence of cytosine methylation in yeast DNA was the first, essential step towards an understanding of the function of this methylation in yeast [23]. Natural epigenetic variation in populations is believed to play a key role in their adaptation to the environment [25]. Organisms interact with their environment, modifying patterns of expression of their genes [26,27]. This can be mediated by various processes. Biotic and abiotic stress factors are stimuli which can cause changes in DNA methylation [25].

The present study investigated the influence of selected physical and chemical factors on the global DNA methylation level in *C. albicans* cells. As reported by other researchers, the degree of methylation depended on the factor and the exposure time [28]. Previous research by Gryzinska et al. [29] on changes in the epigenome of *C. albicans* following ozone exposure showed that the DNA methylation level increased with the exposure time. In the present study, we used different types of radiation (ERM, MR, IR, LASER, UVC, and X-ray) and also showed that the methylation level increased with the duration of exposure to the various types of radiation. The results also showed a significant influence of the type of radiation. The highest methylated DNA level was observed following the exposure of *C. albicans* cells to IR and MR. Infrared is a type of electromagnetic radiation encompassing wavelengths from 780 nm to 1000 μm [30]. Many studies have shown that low-level light therapy (LLLT) or photobiomodulation (PBM) therapy employs light at red and near-infrared wavelengths (600–1000 nm) to modulate biological activity to improve skin wound healing, relieve pain and symptoms of ankylosing spondylitis, and treat cancer and ophthalmological and neurological diseases [30]. Infrared-induced physiological effects are thought to be due to two main types of photoacceptor: cytochrome c oxidase and intracellular water [31]. Photon absorption converts light into signals that can stimulate biological processes [32]. The action of infrared light on water dynamics in membranes, mitochondria, and/or cells could modulate signaling pathways and the production of reactive oxygen species, ATP, Ca^2+^, NO, and inositol phosphates [33,34]. Infrared radiation is an important therapeutic tool. However, our research showed that the use of infrared radiation additionally causes a significant increase in the level of DNA methylation. Radiotherapy has the potential to promote the coevolution of genetic and epigenetic changes. The cellular response to radiation aims to protect the cell, and DNA methylation alterations may define mechanisms mediating cell survival after radiation [35]. Profiling of altered DNA methylation patterns following irradiation may help to identify stress adaptation strategies of cancer cells, as cell programs controlled by epigenetic mechanisms could be one way for cells to resist stress, e.g., to survive radiotherapy [36,37]. In our study, X-ray radiation also caused hypermethylation. After just 10 and 15 min of radiation with X-rays, the percentage of methylated DNA was significantly higher than in cells that were not irradiated. Radiotherapy using X-rays is one of the most effective cancer treatments. Epigenetics, which is widely studied in cancer biology as a mechanism regulating cancer cell biology, deals with the regulation of gene expression primarily through DNA methylation. The treatment of breast cancer cells with X-rays results in extensive changes in DNA methylation. Genes with altered DNA methylation have been found to be enriched with pathways such as DNA repair, apoptosis, and the cell cycle [38]. Changes in DNA methylation are involved in the cell’s defense mechanisms triggered by X-ray exposure. Kim et al. [39] showed that DNA methyltransferase inhibitors radiosensitize human cancer cells by suppressing DNA repair activity, reducing the ability to repair DNA double-strand breaks in cells exposed to radiation. In our research, the treatment of *C. albicans* with laser radiation for a short time (1 and 3 min) caused a decrease in the degree of DNA methylation compared to the control, while longer exposure to laser radiation (5 min) caused an increase in DNA methylation. Laser irradiation is a factor which regulates epigenetic mechanisms [40]. Low-level laser therapy causes changes in cellular mechanisms, including DNA synthesis and gene expression, cell proliferation, and differentiation. The effect of laser irradiation on global DNA methylation and the expression of genes encoding DNA methylotransferases was investigated in a rat model of skin wound healing [41]. A decrease in global DNA methylation was shown, confirming the role of DNA methylation in the healing process. The demethylation of the promoter is accompanied by the activation of specific genes at various stages of the development process. A decrease in the methylation rate, e.g., in embryonic stem cells, could trigger cell orientation to a specific lineage and the expression of differentiation-associated markers [42]. Ultraviolet light (UV) is one of the most DNA-damaging agents in the natural environment and one of the main causes of skin cancer; it can induce basal cell carcinoma, squamous cell carcinoma, and melanoma [43]. UV light is divided into three wavelength bands: UVA (320–400 nm), UVB (290–320 nm), and UVC (100–280 nm) [44]. In the present study, we tested the influence of UVC radiation on the degree of DNA methylation. We showed that longer exposure of *C. albicans* cells to UVC radiation (30 min) resulted in a significant increase in the level of DNA methylation. Leung and Murray [43] investigated the influence of DNA methylation on the level of UVC-induced DNA damage. They showed that DNA methylation significantly decreased the level of DNA damage, as the production of the two major photoproducts, cyclobutane pyrimidine dimer and 6-4 photoproduct adducts, was significantly lower following UVC irradiation compared with the non-methylated analog. We showed an increase in the level of DNA methylation after prolonged UVC irradiation. This indicates that it is a defensive reaction of cells to this environmental factor, as methylated DNA is less sensitive to damage.

In our research, another factor that caused changes in DNA methylation was temperature, which indicates that DNA methylation is dynamic and that abiotic environmental stimuli can lead to changes in methylation. Incubation at both 37 °C and 42 °C resulted in a significant increase in the level of DNA methylation compared to the level at 30 °C. In addition, extending the time of exposure to higher temperatures, especially 42 °C, significantly increased the level of methylation. Changes in DNA methylation have the potential to affect the resilience of species to climate change, including environmental temperature, in both plants [45,46] and animals [47]. Metzger and Schulte [48] assessed the effects of development temperature and adult acclimation temperature on DNA methylation levels in three-spined stickleback (*Gasterosteus aculeatus*) and showed that both increases and decreases in temperature during development and in adults acclimated to the change in temperature increased global DNA methylation levels. This indicates that temperature changes can have long-lasting effects on the epigenome, and that epigenetic modifications in response to temperature change may modulate the capacity of organisms to cope with environmental change.

In our research, we also investigated the influence of a decreased concentration of O_2_, an increased concentration of CO_2_, and anaerobic conditions on global DNA methylation levels in *C. albicans*. Under the conditions of reduced oxygen concentration (5–15% O_2_), the level of DNA methylation increased significantly. On the other hand, the presence of more than 3% CO_2_ or anaerobic culture conditions caused a significant decrease in the degree of DNA methylation. *C. albicans* can exist in various forms, including yeast cells, pseudohyphae, hyphae, and chlamydospores, which are large, round, thick-walled cells with a high lipid content. The growth of *C. albicans* in hyphal form is directly linked to its pathogenicity [49]. *C. albicans* has been observed to produce pseudohyphae under conditions of 10% CO_2_, whereas anaerobic conditions combined with high carbon dioxide content favor the formation of chlamydospores in the absence of nutrient restriction [50]. In these conditions, we observed a decrease in the degree of DNA methylation. Bartelli et al. [51] showed that environmental conditions, such as hypoxia and 37 °C, decrease mitochondrial genome methylation in *C. albicans*. The decrease in DNA methylation levels in *C. albicans* induced by anaerobic conditions may be an epigenetic mechanism affecting adaptation and the pathogenicity of *C. albicans* in human tissues.

## 4. Materials and Methods

*C. albicans* ATCC 10231 was used in this study. The strain was grown in YPD broth (1% *w*/*v* yeast extract, 2% *w*/*v* peptone, 2% *w*/*v* dextrose) (BBL, Becton Dickinson, Sparks, Md., Franklin Lakes, NJ, USA) at 30 °C for 18 h. Then, the yeast cells from the liquid culture were inoculated on agar plates with YPD and incubated at 30 °C for 18 h. Subsequently, the yeast cells were suspended in sterile phosphate-buffered saline (PBS, pH 7.4) to obtain an optical density of OD550 = 0.75 (densitometer DEN-1, Biosan, Rīga, Latvia) (equal to a cell concentration of approx. 2 × 10^7^ CFU/mL), and the suspension was diluted 1:10 (*v*/*v*) in YPD broth. The final concentration of the yeast cells was about 10^6^ CFU/mL and was used for all experiments.

*C. albicans* ATCC 10231 cells were subjected to methylation stress using three types of physical and chemical factors: radiation, temperature, and controlled atmosphere.

### 4.1. Testing of the Influence of Radiation on the Degree of Cytosine Methylation in DNA

A 10 mL volume of the yeast cell suspension with about 10^6^ CFU/mL in YPD broth was transferred into a sterile Petri dish and treated with various types of radiation. The time of exposure to each type of radiation is shown in Table 2.

#### 4.1.1. Electromagnetic Radiation (EMR)

Petri dishes with suspensions of yeast cells were placed 3 m from a transformer station (Trafo, Lublin, Poland) emitting EMR with voltage of 15 kV/0.4 kV.

#### 4.1.2. Microwave Radiation (MR)

A 2450 MHz microwave oven (LG) with electrical power of 900 W was used. Petri dishes with a yeast cell suspension were placed on the microwave plate.

#### 4.1.3. Infrared Radiation (IR)

Petri dishes with a yeast cell suspension were placed 20 cm from a Sollux Lumina V5.0 lamp with radiator power of 375 W.

#### 4.1.4. Light Amplification by Stimulated Emission of Radiation (LASER)

A diode laser (Oriel, Orroyo Instruments, LTIO00-PLT20, NLC, Pretoria, South Africa) was used. Cell suspensions were exposed to the laser at a wavelength of 636 nm in the dark. Laser radiation was delivered to the cell suspension using an optical cable. The laser parameters were as follows: wavelength 636 nm, power output 85 mW, spot size 9.08 cm^2^, output density 9.3 mW/cm^2^, fluence 5 J/cm^2^, wave emission—continuous wave. The laser did not increase the temperature of the suspension above 1 °C.

#### 4.1.5. UVC Radiation

A 10 mL volume of cell suspension was transferred into sterile Petri dishes and placed on a table 2 m from an NBV 30 NL germicidal lamp (Ultraviol, Zgierz, Poland), which emitted light at 253.7 nm. The suspensions were perpendicular to the UVC source (horizontal) and directly exposed to UVC.

#### 4.1.6. X-ray Radiation

The cell suspension was irradiated with X-rays using a Rad Source RS-2000 Biological Irradiator. X-rays were emitted at 2 Gy/min, at a distance of 20 cm.

In each test of the effects of radiation, a second series of suspensions containing the same amount of *C. albicans* ATCC 10231 cells, which were not exposed to radiation, were used as controls. Each assay was performed in triplicate, and the results were averaged. After each time point, *C. albicans* ATCC 10231 genomic DNA was extracted.

### 4.2. Testing of the Influence of Temperature on the Degree of Cytosine Methylation in DNA

Suspensions of yeast cells in YPD broth (about 10^6^ CFU/mL) were transferred to tubes (10 mL per tube) and incubated at 37 °C and 42 °C for 90, 180, or 360 min. Suspensions containing the same amount of yeast cells, incubated in tubes at 30 °C for the same time periods, were used as controls. After each time point, *C. albicans* ATCC 10231 genomic DNA was extracted. Each assay was performed in triplicate, and the results were averaged.

### 4.3. Testing of the Influence of Controlled Atmosphere on the Degree of Cytosine Methylation in DNA

*C. albicans* ATCC 10231 cells from liquid culture were inoculated on agar plates with YPD and incubated at 30 °C for 24 h, at >3% CO_2_ (BD GasPakTM EZ CO_2_ Gas Generating Pouch System), at 5–15% O_2_ (BD GasPakTM EZ Campy Gas Generating Pouch System), and in anaerobic conditions (10% H_2_, 10% CO_2_, and 80% N_2_ as the gas mixture) (BD GasPakTM EZ Anaerobe Gas Generating Pouch System with indicator) (Becton, Dickinson and Company, 7 Loveton Circle, Sparks, ML, 21152, USA). Cultures of *C. albicans* ATCC 10231 carried out in aerobic conditions at 30 °C for 24 h were used as controls. Then, *C. albicans* ATCC 10231 genomic DNA was extracted. Each assay was performed in triplicate, and the results were averaged.

### 4.4. Isolation of DNA from C. albicans ATCC 10231 Cells and DNA Methylation Determination

DNA was isolated using the NucleoSpin^®^ Plant II kit (Macherey-Nagel). DNA was isolated from 81 *C. albicans* ATCC 10231 samples exposed to various factors in different treatment times and from 81 *C. albicans* ATCC 10231 samples that were not exposed to these factors (control samples). The total DNA methylation level was determined using a kit for the quantitative analysis of DNA methylation—the Imprint Methylated DNA Quantification Kit (MDQ1) (Sigma Aldrich, Steinheim, Germany). The detection limit of the used kit is 5 ng of fully methylated DNA per 200 ng of total DNA amount tested (2.5%). In order to assess the level of methylated DNA in *C. albicans* ATCC 10231 cells that were exposed to the tested factors and the level of methylated DNA in cells that were not treated with these factors, 96-well plates were used. The DNA samples were diluted in binding solution to give a final concentration of 150 ng/µL. The diluted DNA samples (30 µL) were transferred to wells and incubated at 37 °C for 1 h to bind DNA. Then, block solution was added, and samples were incubated again at 37 °C for 30 min. After that, methylated DNA was captured using diluted capture antibodies and detected by binding to the previously diluted detection antibodies. After the addition of the developing solution and incubation (10 min), the stop solution was added (the solution changed color from blue to yellow). Both a blank control and a methylated control (positive control) were analyzed together with the DNA samples. The absorbance was measured at 450 nm. The global DNA methylation level was established as the methylation level in the samples relative to the methylated control.

The DNA methylation level was calculated according to the following equation:

[(A450S − A450B)/(A450MC − A450B)] × 100(1)

where:

A450S—average absorbance of the sample;
A450B—average absorbance of the blank;
A450MC—average absorbance of the methylated control.

### 4.5. Statistical Analysis

Calculations were performed for each factor and exposure time using the mean of three measurements. Statistical analysis of the results (Duncan’s test) was performed using SAS 9.4 software. The significance of differences was determined at *p* ≤ 0.05.

## 5. Conclusions

Our study assessing the effect of physical and chemical factors on DNA methylation is in line with current research identifying physical factors adversely affecting the functioning of organisms. Various types of radiation and their little-known effects on organisms can pose a threat to their functioning. In the present study, we used the reference strain *C. albicans* ATCC 10231. We showed that longer exposure to various types of radiation significantly increased the level of methylated DNA, which indicates that the rapidly increasing attachment of methyl groups to cytosine bases accompanying the prolonged exposure of DNA to radiation is linked to the enhanced activity of methyltransferases. This reaction may be linked to the protection of DNA against damage but can also lead to epigenetic disorders and thus adversely affect the functioning of organisms. We also showed that elevated temperature above the optimal temperature for the growth of *C. albicans* is a factor increasing the methylation level. Reduced content of O_2_ in the growth atmosphere for *C. albicans* caused a significant increase in the DNA methylation level. In contrast, the level of methylated DNA decreased in anaerobic conditions and in conditions of increased CO_2_ concentration, which may indicate that epigenetic processes enable the adaptation of *C. albicans* to the conditions prevailing in the host organism. It is necessary to conduct further research assessing the effects of factors that caused the greatest increase in methylation levels, in order to check their impact on the methylation level and the expression of genes such as oncogenes or genes related to chronic inflammation, which could be helpful in finding a factor with potential as a therapeutic tool.

## Figures and Tables

**Table 1:** DNA methylation levels in *Candida albicans* ATCC 10231 exposed to various physical and chemical factors.

Factor Type	Factor ^1^	Exposure Time	Global DNA Methylation (%)	
			Control Samples	Experimental Samples
Radiation	EMR	24 h	52.23 ± 0.60	51.49 ^b^ ± 0.52
		48 h	52.87 ± 1.08	52.85 ^b^ ± 0.55
		72 h	53.32 ± 1.51	57.08 ^a,^* ± 0.31
	MR	15 s	50.34 ± 0.08	56.18 ± 0.68
		30 s	50.57 ± 0.19	57.80 ± 0.33
		45 s	50.60 ± 0.23	62.75 * ± 6.24
	IR	5 min	50.60 ± 0.49	53.59 ^c,^* ± 0.70
		15 min	51.04 ± 0.48	56.68 ^b,^* ± 0.88
		30 min	51.56 ± 0.87	64.36 ^a,^* ± 0.88
	LASER	1 min	50.63 ± 0.18	48.39 ^b,^* ± 0.88
		3 min	50.65 ± 0.18	49.50 ^b^ ± 0.98
		5 min	50.76 ± 0.09	53.84 ^a,^* ± 0.98
	UVC	10 min	50.65 ± 0.46	50.74 ^b^ ± 0.98
		20 min	50.96 ± 0.07	51.24 ^b^ ± 0.98
		30 min	51.56 ± 0.87	55.57 ^a,^* ± 0.98
	X-ray	5 min	50.60 ± 0.49	55.07 ± 0.98
		10 min	50.65 ± 0.46	58.79 * ± 0.98
		15 min	51.04 ± 0.48	61.14 * ± 5.81
Temperature	37 °C	90 min	51.07 ± 0.46	54.95 ^b,^* ± 0.98
		180 min	51.36 ± 0.64	56.68 ^b,^* ± 0.98
		360 min	51.42 ± 1.24	61.51 ^a,^* ± 1.53
	42 °C	90 min	51.07 ± 0.46	57.67 ^c,^* ± 0.98
		180 min	51.36 ± 0.64	63.00 ^b,^* ± 1.53
		360 min	51.42 ± 1.24	69.55 ^a,^* ± 1.53
Controlled atmosphere	5–15% O_2_, 30 °C	24 h	52.23 ± 0.60	57.56 ^a,^* ± 6.48
	>3% CO_2_, 30 °C	24 h	52.23 ± 0.60	49.68 ^b^ ± 3.55
	(10% H_2_, 10% CO_2_, 80% N_2_) **, 30 °C	24 h	52.23 ± 0.60	50.23 ^b^ ± 1.31

^1^ EMR—electromagnetic radiation, MR—microwave radiation, IR—infrared radiation, LASER—Light Amplification by Stimulated Emission of Radiation; UVC—ultraviolet radiation in a wavelength range of 100–280 nm (C); X-ray—roentgen radiation. ^a^, ^b^, ^c^—means columns for a given factor with different superscript letters are significantly different at *p* ≤ 0.05 (Duncan’s test). *—means rows for the experimental and control samples marked with an asterisk are significantly different at *p* ≤ 0.05 (Duncan’s test). ** Anaerobic conditions. Each assay was performed three times, and the results were averaged.

**Table 2:** Type of physical and chemical factors and exposure time.

Factor Type	Factor *	Exposure Time
Radiation	EMR	24, 48, 72 h
	MR	15, 30, 45 s
	IR	5, 15, 30 min
	LASER	1, 3, 5 min
	UVC	10, 20, 30 min
	X-ray	5, 10, 15 min
Temperature	37 °C	90, 180, 360 min
	42 °C	90, 180, 360 min
Controlled atmosphere	5–15% O_2_, 30 °C	24 h
	>3% CO_2_, 30 °C	24 h
	(10% H_2_, 10% CO_2_ and 80% N_2_) **, 30 °C	24 h

* EMR—electromagnetic radiation, MR—microwave radiation, IR—infrared radiation, LASER—Light Amplification by Stimulated Emission of Radiation; UVC—ultraviolet radiation in a wavelength range of 100–280 nm (C); X-ray—roentgen radiation. ** Anaerobic conditions.

## Data Availability

All representative data are included in the manuscript.

## References

[1] Crisp P.A., Ganguly D., Eichten S.R., Borevitz J.O., Pogson B.J. (2016). Reconsidering plant memory: Intersections between stress recovery, RNA turnover, and epigenetics. Sci. Adv..

[2] Verhoeven K.J.F., vonHoldt B.M., Sork V.L. (2016). Epigenetics in ecology and evolution: What we know and what we need to know. Mol. Ecol..

[3] Ho D.H., Burggren W.W. (2010). Epigenetics and transgenerational transfer: A physiological perspective. J. Exp. Biol..

[4] Moen E.L., Mariani C.J., Zullow H., Jeff-Eke M., Litwin E., Nikitas J.N., Godley L.A. (2015). New themes in the biological functions of 5-methylcytosine and 5-hydroxymethylcytosine. Immunol. Rev..

[5] Wilson A.S., Power B.E., Molloy P.L. (2007). DNA hypomethylation and human diseases. Biochim. Biophys. Acta.

[6] Li E., Zhang Y. (2014). DNA methylation in mammals. Cold Spring Harb. Perspect. Biol..

[7] Hattman S., Kenny C., Berger L., Pratt K. (1978). Comparative study of DNA methylation in three unicellular eucaryotes. J. Bacteriol..

[8] Gryzinska M., Andraszek K., Jezewska-Witkowska G. (2014). Estimation of Global Content of 5-methylcytosine in DNA during Allantoic and Pulmonary Respiration in the Chicken Embryo. Folia Biol..

[9] Gryzinska M., Błaszczak E., Strachecka A., Jeżewska-Witkowska G. (2013). Analysis of Age-Related Global DNA Methylation in Chicken. Biochem. Genet..

[10] Gryzinska M., Jakubczak A., Listos P., Dudko P., Abramowicz K., Jeżewska-Witkowska G. (2016). Association between body weight and age of dogs and global DNA methylation. Med. Weter.

[11] Andraszek K., Gryzinska M., Wójcik E., Knaga S., Smalec E. (2014). Age-dependent change in the morphology of nucleoli and methylation of genes of the nucleolar organizer region in the Japanese quail model *Coturnix japonica* (Temminck and Schlegel, 1849) (Galliformes: Aves). Folia Biol..

[12] Lan H., Sun R., Fan K., Yang K., Zhang F., Nie X.Y., Wang X., Zhuang Z., Wang S. (2016). The Aspergillus flavus Histone Acetyltransferase Afl GcnE Regulates Morphogenesis, Aflatoxin Biosynthesis, and Pathogenicity. Front. Microbiol..

[13] Martienssen R.A., Colot V. (2001). DNA methylation and epigenetic inheritance in plants and filamentous fungi. Science.

[14] Weil C., Martienssen R. (2008). Epigenetic interactions between transposons and genes: Lessons from plants. Curr. Opin. Genet. Dev..

[15] Aparicio J.G., Viggiani C.J., Gibson D.G., Aparicio O.M. (2004). The Rpd3-Sin3 histone deacetylase regulates replication timing and enables intra-S origin control in *Saccharomyces cerevisiae*. Mol. Cell. Biol..

[16] Law J.A., Jacobsen S.E. (2010). Establishing, maintaining and modifying DNA methylation patterns in plants and animals. Nat. Rev. Genet..

[17] Volpe T.A. (2002). Regulation of Heterochromatic Silencing and Histone H3 Lysine-9 Methylation by RNAi. Science.

[18] Mayer F.L., Wilson D., Hube B. (2013). *Candida albicans* pathogenicity mechanisms. Virulence.

[19] Czechowicz P., Nowicka J., Gościniak G. (2022). Virulence Factors of *Candida* spp. and Host Immune Response Important in the Pathogenesis of Vulvovaginal Candidiasis. Int. J. Mol. Sci..

[20] Huang G. (2012). Regulation of phenotypic transitions in the fungal pathogen *Candida albicans*. Virulence.

[21] Hirakawa M.P., Martinez D.A., Sakthikumar S., Anderson M., Berlin S., Gujja S., Zeng Q., Zisson E., Wang J.M., Greenberg J.M. (2015). Genetic and phenotypic intra-species variation in *Candida albicans*. Genome Res..

[22] Whiteway M., Bachewich C. (2007). Morphogenesis in *Candida albicans*. Annu. Rev. Microbiol..

[23] Tang Y., Gao X.D., Wang Y., Yuan B.F., Feng Y.Q. (2012). Widespread Existence of Cytosine Methylation in Yeast DNA Measured by Gas Chromatography/Mass Spectrometry. Anal. Chem..

[24] Turchetti B., Marconi G., Sannino C., Buzzini P., Albertini E. (2020). DNA Methylation Changes Induced by Cold in Psychrophilic and Psychrotolerant *Naganishia* Yeast Species. Microorganisms.

[25] Sahu P.P., Pandey G., Sharma N., Puranik S., Muthamilarasan M., Prasad M. (2013). Epigenetic mechanisms of plant stress responses and adaptation. Plant Cell Rep..

[26] Yaish M.W., Colasanti J., Rothstein S.J. (2011). The role of epigenetic processes in controlling flowering time in plants exposed to stress. J. Exp. Bot..

[27] Richards C.L., Alonso C., Becker C., Bossdorf O., Bucher E., Colomé-Tatché M., Durka W., Engelhardt J., Gaspar B., Gogol-Doring A. (2017). Ecological plant epigenetics: Evidence from model and non-model species, and the way forward. Ecol. Lett..

[28] Martin E.M., Fry R.C. (2018). Environmental Influences on the Epigenome: Exposure Associated DNA Methylation in Human Populations. Annu. Rev. Public Health.

[29] Gryzińska M., Wlazło Ł., Nowakowicz-Dębek B., Jeżewska-Witkowska G., Jakubczak A. (2019). DNA methylation in yeast-like fungi of the species *Candida albicans* induced by different lengths of exposure to ozone. Rus. J. Genet..

[30] Tsai S.R., Hamblin M.R. (2017). Biological effects and medical applications of infrared radiation. J. Photochem. Photobiol. B.

[31] Passarella S., Karu T. (2014). Absorption of monochromatic and narrow band radiation in the visible and near IR by both mitochondrial and non-mitochondrial photoacceptors results in photobiomodulation. J. Photochem. Photobiol. B.

[32] Bashkatov A.N., Genina E.A., Kochubey V.I., Tuchin V.V. (2005). Optical properties of human skin, subcutaneous and mucous tissues in the wavelength range from 400 to 2000 nm. J. Phys. D Appl. Phys..

[33] Irvine R.F., Schell M.J. (2001). Back in the water: The return of the inositol phosphates. Nat. Rev. Mol. Cell Biol..

[34] de Freitas L.F., Hamblin M.R. (2016). Proposed Mechanisms of Photobiomodulation or Low-Level Light Therapy. IEEE J. Sel. Top. Quantum Electron..

[35] Danielsson A., Barreau K., Kling T., Tisell M., Carén H. (2020). Accumulation of DNA methylation alterations in paediatric glioma stem cells following fractionated dose irradiation. Clin. Epigenet..

[36] Dabin J., Fortuny A., Polo S.E. (2016). Epigenome maintenance in response to DNA damage. Mol. Cell..

[37] Zhu X., Wang Y., Tan L., Fu X. (2018). The pivotal role of DNA methylation in the radio-sensitivity of tumor radiotherapy. Cancer Med..

[38] Antwih D.A., Gabbara K.M., Lancaster W.D., Ruden D.M., Zielske S.P. (2013). Radiation-induced epigenetic DNA methylation modification of radiation-response pathways. Epigenetics.

[39] Kim H.J., Kim J.H., Chie E.K., Young P.D., Kim I.A., Kim I.H. (2012). DNMT (DNA methyltransferase) inhibitors radiosensitize human cancer cells by suppressing DNA repair activity. Radiat. Oncol..

[40] Zamani A.R.N., Saberianpour S., Geranmayeh M.H., Bani F., Haghighi L., Rahbarghazi R. (2020). Modulatory effect of photobiomodulation on stem cell epigenetic memory: A highlight on differentiation capacity. Lasers Med. Sci..

[41] Gomes M.V.d.M., Manfredo M.H., Toffoli L.V., Castro-Alves D.C., Lucas Magnoni do Nascimento L., da Silva W.R., Kashimoto R.K., Rodrigues G.M., Estrada V.B., Andraus R.A. (2016). Effects of the led therapy on the global DNA methylation and the expression of Dnmt1 and Dnmt3a genes in a rat model of skin wound healing. Lasers Med. Sci..

[42] Kim M., Costello J. (2017). DNA methylation: An epigenetic mark of cellular memory. Exp. Mol. Med..

[43] Leung W.Y., Murray V. (2021). The influence of DNA methylation on the sequence specificity of UVB- and UVC-induced DNA damage. J. Photochem. Photobiol. B.

[44] de Oliveira N.F.P., de Souza B.F., de Castro Coêlho M. (2020). UV Radiation and Its Relation to DNA Methylation in Epidermal Cells: A Review. Epigenomes.

[45] Syngelaki E., Schinkel C.C.F., Klatt S., Hörandl E. (2020). Effects of Temperature Treatments on Cytosine-Methylation Profiles of Diploid and Autotetraploid Plants of the Alpine Species *Ranunculus kuepferi* (Ranunculaceae). Front. Plant Sci..

[46] Naydenov M., Baev V., Apostolova E., Gospodinova N., Sablok G., Gozmanova M., Yahubyan G. (2015). High-temperature effect on genes engaged in DNA methylation and affected by DNA methylation in *Arabidopsis*. Plant Physiol. Biochem..

[47] Lallias D., Bernard M., Ciobotaru C., Dechamp N., Labbé L., Goardon L., Le Calvez J.M., Bideau M., Fricot A., Prézelin A. (2021). Sources of variation of DNA methylation in rainbow trout: Combined effects of temperature and genetic background. Epigenetics.

[48] Metzger D.C.H., Schulte P.M. (2017). Persistent and plastic effects of temperature on DNA methylation across the genome of threespine stickleback (*Gasterosteus aculeatus*). Proc. Biol. Sci..

[49] Noble S.M., Gianetti B.A., Witchley J.N. (2017). *Candida albicans* cell type switches and functional plasticity in the mammalian host. Nat. Rev. Microbiol..

[50] Williams S., Cleary I., Thomas D. (2023). Anaerobic conditions are a major influence on *Candida albicans* chlamydospore formation. Folia Microbiol..

[51] Bartelli T.F., Bruno D.C.F., Briones M.R.S. (2018). Evidence for Mitochondrial Genome Methylation in the Yeast *Candida albicans*: A Potential Novel Epigenetic Mechanism Affecting Adaptation and Pathogenicity?. Front. Genet..

